# Biomechanical risk factors for tibial plateau fracture following Oxford unicompartmental knee arthroplasty: a finite element analysis under gait loading

**DOI:** 10.3389/fbioe.2026.1799341

**Published:** 2026-05-21

**Authors:** Jiazhong Ji, Long Xue, Yue Hou, Zhaoyang Li, Yihui Tu, Huaming Xue, Tao Wen, Tao Yang, Tong Ma

**Affiliations:** 1 Department of Orthopaedics, Yangpu Hospital, School of Medicine, Tongji University, Shanghai, China; 2 Department of Gynecology, Jinan Maternity and Child Care Hospital Affiliated to Shandong First Medical University, Jinan, China

**Keywords:** biomechanics, finite element analysis, gait loading, Oxford prosthesis, tibial plateau fracture, unicompartmental knee arthroplasty

## Abstract

**Background:**

Tibial plateau fractures following Oxford unicompartmental knee arthroplasty (UKA), though rare (<1% incidence), represent serious postoperative complications that often necessitate complex revision surgery. This study aimed to systematically investigate the biomechanical risk factors for tibial plateau fractures following Oxford UKA by evaluating the effects of prosthesis positioning parameters on tibial stress distribution under dynamic gait loading.

**Methods:**

Ten distinct models were analyzed under ISO-standardized gait cycle loading to assess the impact of coronal alignment angles (−7°, −3°, 0°, 3°, 7°), sagittal bone cut (1 mm, 3 mm, 8 mm), and femoral-tibial prosthesis positioning (3 mm, 5 mm shifts). Maximum von-Mises stress on the tibial cortex was measured, with particular focus on the posterior medial cortex. Both von Mises stress and Maximum Principal Stress were evaluated to assess general equivalent loads and specific tensile fracture risks, respectively.

**Results:**

In the neutral position, maximum stress measured 19.01 MPa post-gait loading. Varus positioning at −3° yielded the minimum stress (18.06 MPa), while 7° valgus resulted in dramatically elevated stress (48.15 MPa, 2.5-fold increase). Longitudinal sagittal bone cut significantly influenced stress distribution: 1 mm over-cut increased stress to 28.21 MPa, 3 mm to 29.23 MPa, and 8 mm depth reached 45.10 MPa (2.3-fold increase). Femoral prosthesis displacement of 5 mm relative to the tibial component elevated posterior cortex stress to 33.24 MPa (1.7-fold increase from neutral). Notably, eversion-induced stress increases exceeded those from inversion, and these parameters exhibited nonlinear interactions. Principal stress analysis revealed that severe valgus alignment (7°) and deep sagittal cuts (8 mm) drastically concentrated tensile stresses at the posterior tibial cortex, peaking at 15.1 MPa and 12.56 MPa, respectively.

**Conclusion:**

Prosthesis malalignment (particularly excessive valgus beyond 3°) and depth of tibial sagittal osteotomy (>3 mm) are primary contributors to elevated tibial stress. Based on these biomechanical findings, we recommend that the Oxford unicompartmental prosthesis should be positioned at 3° varus in the coronal plane, the femoral-tibial prosthesis distance should not exceed 3 mm, and the longitudinal sagittal bone cut should be limited to 3 mm depth.

## Introduction

Knee osteoarthritis remains a primary driver of global disability and functional impairment, with recent estimates from the Global Burden of Disease Study indicating that it affects more than 527 million people worldwide (Safiri et al., 2020). For patients whose osteoarthritis is confined to a single compartment, unicompartmental knee arthroplasty (UKA) has emerged as a significant alternative to total knee arthroplasty. This procedure offers numerous advantages, including short operative time, accelerated recovery, and superior preservation of native knee joint kinematics (Jennings et al., 2019). Advances in surgical techniques and prosthetic design have substantially improved the 10-year survival rate of UKA to over 94%, demonstrating excellent long-term clinical outcomes in carefully selected patients (Pandit et al., 2015).

Despite these advances, postoperative complications continue to significantly impact the success rate of UKA. Among these, tibial plateau fractures, although rare, represent serious complications with an incidence rate of less than 1% as reported in the literature (Ji et al., 2014; Burger et al., 2022; Kim et al., 2016; Mohammad et al., 2020; Brown et al., 2019). These fractures typically occur within months after surgery, causing severe pain and functional impairment, and often necessitate complex revision surgery (Wood et al., 2024). Despite their low incidence, the significant impact of tibial plateau fractures on patient prognosis and the rising number of UKA surgeries have heightened attention to this issue (Burger et al., 2022; Wood et al., 2024).

The pathogenesis of tibial plateau fractures following UKA is complex, involving both patient-related factors, such as osteoporosis, body mass index, and age, and surgical technique factors (Campi et al., 2018; Yokoyama et al., 2019). Biomechanically, improper prosthesis placement is considered the primary cause of stress concentration and abnormal load distribution, which heightens fracture risk (Innocenti et al., 2016). Specifically, the coronal varus and valgus alignment after unicompartmental surgery, the depth of the tibial plateau’s longitudinal section, and the positional relationship between prostheses can significantly impact tibial stress distribution. Studies indicate that excessive coronal valgus post-surgery can increase tibial cortical stress and fracture risk (Innocenti et al., 2016; Kang et al., 2018a; Inoue et al., 2016). However, there is no consensus on the optimal alignment angle, with recommended values differing across research reports. The depth of the longitudinal incision on the tibial side during surgery significantly affects the load-bearing capacity and stress distribution at the prosthesis-bone interface. However, there is limited evidence on its quantitative relationship with fracture risk. Additionally, despite being one of the most widely used mobile platform prosthesis systems, the positional relationship between the femoral and tibial prostheses in the Oxford UKA has not been thoroughly examined for its impact on contact mechanics and stress transmission pathways.

Finite element analysis (FEA) has proven to be an effective tool for studying orthopedic biomechanical problems, allowing for the quantitative evaluation of how different surgical parameters affect bone stress distribution under non-invasive conditions (Carr and Goswami, 2009; Eghan-Acquah et al., 2025; Awal et al., 2025). Although some researchers have used the finite element method to examine the biomechanical properties of UKA (Innocenti et al., 2016), most studies have focused on evaluating prosthesis design or predicting polyethylene wear, primarily using static or simplified loading conditions (Small et al., 2013). In actual physiological activities, the knee joint experiences complex dynamic loads, particularly during the gait cycle, where both the magnitude and direction of the loads constantly change (Affatato, 2016; Shim et al., 2010). Therefore, systematically assessing the impact of various surgical technical parameters on the risk of tibial plateau fractures under physiological loading conditions remains crucial.

Currently, a notable gap exists in systematic research evaluating how coronal varus angle, Oxford unicompartmental condyle position, tibial longitudinal depth, and femur-tibial prosthesis alignment affect stress distribution on the tibial plateau during gait loading. The interactions among these factors and their individual contributions remain uncertain, limiting clinicians’ ability to optimize surgical techniques and develop personalized surgical plans. This study aims to develop a high-precision three-dimensional finite element model incorporating the Oxford unicompartmental prosthesis to simulate physiological gait loading conditions. Through this model, we systematically assess how the prosthesis’s coronal varus angle, tibial longitudinal incision depth, and femur-tibial prosthesis alignment impact stress distribution on the tibial plateau and fracture risk. This parametric analysis will provide a quantitative biomechanical foundation for optimizing clinical surgical techniques and improving patient outcomes.

## Materials and methods

### Modeling of the healthy and MB UKA knee joint

This study enrolled a healthy 38-year-old male volunteer with a height of 173 cm and body weight of 70 kg. The selection of a 38-year-old healthy male volunteer was intended to establish a standardized anatomical environment with normal bone geometry and quality. This approach minimizes the interference of pre-existing pathological deformities or severe bone loss commonly observed in the elderly population, which could confound the biomechanical response to variations in implant alignment. The participant had no history of lower extremity pathologies, including tumors, degenerative joint disease, infection, or trauma. The participant provided informed consent for imaging examination. The Oxford third-generation removable pad prosthesis (Biomet, Warsaw, IN, United States) was chosen, with a medium-sized femoral prosthesis, a C-sized tibial prosthesis, and a medium-sized/4 mm removable pad. A total of 229^−ΔΔCT^ images were acquired at 0.70 mm intervals, alongside 30 MRI images at 0.56 mm intervals, both on the horizontal plane, using CT and 3.0 T MRI. The images had a resolution of 512 × 512 pixels. CT images were processed to extract bone contour curves and construct a three-dimensional bone model. MRI images were utilized to develop three-dimensional models of soft tissues, including femoral cartilage and the meniscus. Using Mimics software, point cloud matching was employed to assemble the bones and soft tissues, creating a non-destructive knee joint model. The lower ends of the tibia and fibula were secured, and a 1000 N axial load was applied downward along the y-axis at the center points of the femoral medial and lateral condyles (Moglo and Shirazi-Adl, 2003). This 1000 N static load, corresponding to roughly 1.45 times the subject’s body weight, was specifically selected as a standardized benchmark for model validation. This methodology is widely supported by recent established finite element analyses of unicompartmental knee arthroplasty, enabling a robust and comparable assessment of baseline joint contact pressures before the application of dynamic gait cycles (Kang et al., 2018a; Inoue et al., 2016). Upon completing the numerical simulation, the peak contact pressure between the tibial and femoral cartilage was determined and analyzed under various loading conditions. Additionally, the model’s response to the anterior drawer force on the tibia was assessed. The knee joint was positioned in full extension at 30°, 60°, and 90°, and a 130 N load was applied posterior to the femur to simulate the anterior drawer load experiment. Subsequently, the simulated tibial anterior displacement distances were calculated and compared with previously reported experimental data (Suggs et al., 2003).

**FIGURE 1:**
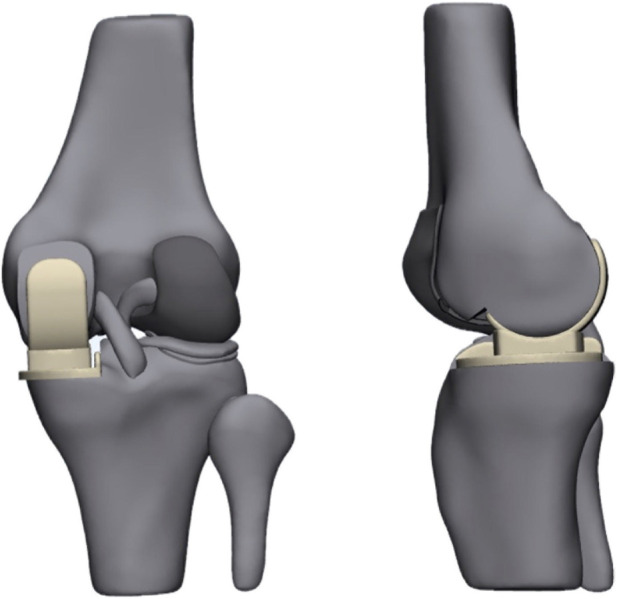
3D model of mobile-bearing UKA.

In the verified complete knee joint model, the prosthesis was implanted into the three-dimensional finite element model following the Oxford III operation guide (Figure 1). Cartilage and meniscus were characterized as isotropic linear elastic materials. Ligaments were modeled as incompressible transversely isotropic superelastic materials. Both the femoral prosthesis and the tibial baseplate were modeled as linearly elastic isotropic materials (Cobalt-chromium alloy). The unicompartmental insert was composed of ultra-high molecular weight polyethylene (UHMWPE) and was also treated as an isotropic linearly elastic material to ensure numerical stability during the dynamic gait cycle simulation (Carr and Goswami, 2009; Galbusera et al., 2014) (Table 1).

**TABLE 1:** Material properties in the finite element models.

Variable	Young’s modulus (MPa)	Poisson’s ratio
Cortical bone	12,000	0.3
Cancellous bone	350	0.25
Cartilage	15	0.46
Meniscus	28	0.33
CoCrMo alloy	195,000	0.3
UHMWPE	685	0.4

CoCrMo alloy, Cobalt–chromium–molybdenum ally; UHMWPE, ultra-high molecular weight polyethylene.

### Finite element mesh and convergence study

The models were meshed utilizing 4-node linear tetrahedral elements (C3D4). To ensure numerical stability, a mesh convergence study was conducted on the tibial cortex by evaluating three global mesh sizes: 2.0 mm, 1.5 mm, and 1.0 mm. Under neutral loading, the maximum von Mises stresses were 59.89 MPa, 61.38 MPa, and 62.43 MPa, respectively. The relative difference between the 1.5 mm and 1.0 mm meshes was approximately 1.7%, which is well within the accepted 5% threshold for convergence. Therefore, the 1.5 mm mesh density (comprising 86,216 elements and 25,270 nodes for the tibial cortex) was adopted for all subsequent simulations. Furthermore, mesh quality was verified using standard metrics, ensuring Jacobian and aspect ratios remained within acceptable limits. This study employed a dynamic analysis solved via the Abaqus/Explicit solver to effectively capture the time-dependent mechanical responses during the standardized gait cycle.

### Loading and boundary conditions

**FIGURE 2:**
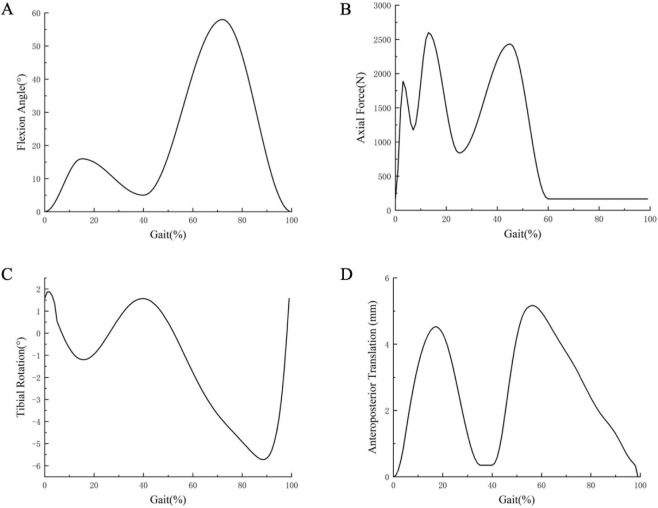
FE model inputs as a function of the gait cycle: **(A)** flexion angle, **(B)** axial load, **(C)** tibial rotation, **(D)** anteroposterior translation.

Loads were applied to the geometric center of the femur following the gait cycle guidelines established by the International Organization for Standardization (ISO). The vertical load was controlled through the up-and-down displacement of the femoral component, while the motion degrees of freedom for other components, except the femoral component, were restricted. The anteroposterior displacement and internal and external rotation of the tibia were controlled by their respective amplitudes, allowing internal and external movement while limiting other motion degrees (Kwon et al., 2014). Abaqus 6.13 software was employed to simulate one gait cycle, and the analysis was performed under the ISO 14243-3 standard. Four curves were incorporated into the model to replicate gait cycles, including flexion and extension, forward and backward translation, axial force, and tibial rotation (Koh et al., 2019) (ISO 141243-3, 2014) (Figure 2). The maximum von-Mises stress was assessed in the medial tibial cortex.

### Simulation of prosthesis malalignment in UKA

**FIGURE 3:**
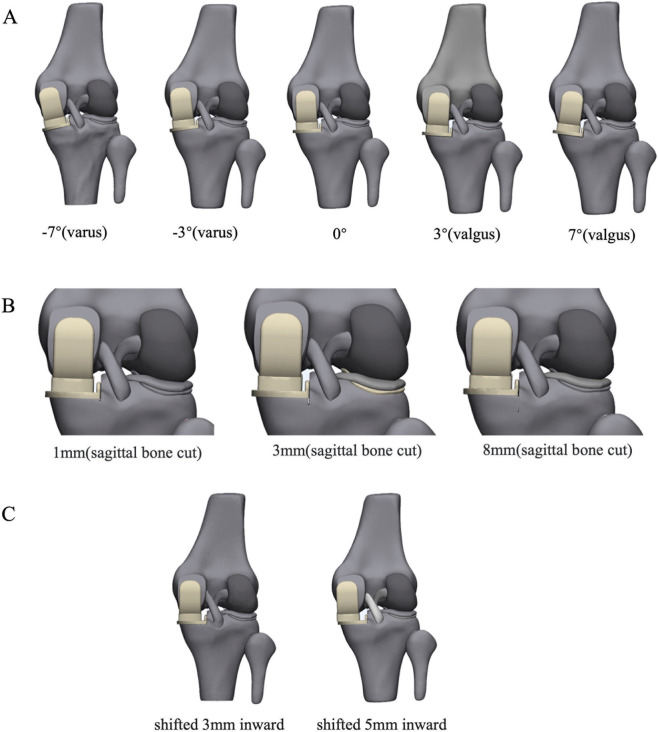
Finite element analysis of implant alignment variations in unicompartmental knee arthroplasty. **(A)** Coronal plane alignment configurations of the femoral component in the UKA finite element model: -7° varus, −3° varus, neutral (0°), 3° valgus,and 7° valgus alignment. **(B)** Depth of tibial over-resection relative to the planned resection level: 1 mm, 3 mm, and 8 mm excessive resection. **(C)** Mediolateral displacement of the femoral component relative to the tibial implant: 3 mm medial offset, and 5 mm medial offset.

**FIGURE 4:**
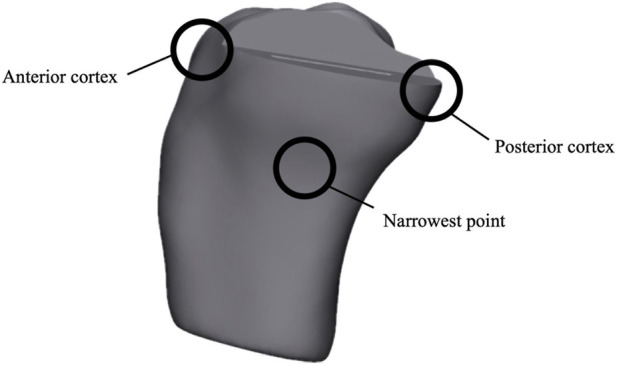
Regional distribution of maximum Von Mises stress in the tibial cortical bone: anterior cortex, posterior cortex and narrowest point.

This study evaluated 10 distinct models, including unicompartmental models with varying coronal angles (−7°, −3°, 0°, 3°, 7°), unicompartmental models with different sagittal tibial transections (1 mm, 3 mm, 8 mm), and unicompartmental models with differing distances between the femoral and tibial prostheses (3 mm, 5 mm) (Figure 3). The research aimed to investigate how various surgical methods affect tibial plateau fractures, focusing primarily on the bone stress of the tibial cortex. Based on previous literature (Simpson et al., 2009), three statistical regions were identified in the cortical bone: the anterior cortex, the posterior cortex, and the tibial narrowest point (Figure 4). In this study, ten models underwent simulation with applied gait loads, and the Von Mises stress of the tibial cortical bone under these loads was measured. To comprehensively evaluate the fracture risk of the anisotropic tibial bone, Maximum Principal Stress was extracted as an additional failure criterion. While von Mises stress provides a macroscopic overview of the equivalent structural load, Maximum Principal Stress specifically identifies localized tensile stress, which is the primary biomechanical driver of crack initiation and split fractures in cortical bone. Negative principal stress values were utilized to indicate compressive states.

## Result

### Model validation

**FIGURE 5:**
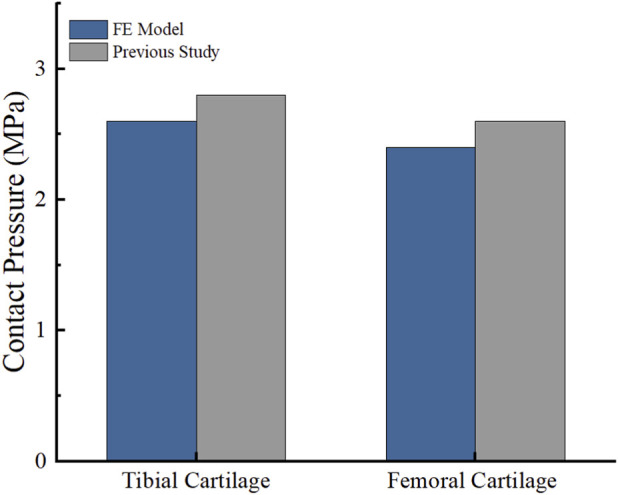
Comparison between previous studies and the present study: femoral and tibial contact pressure.

**FIGURE 6:**
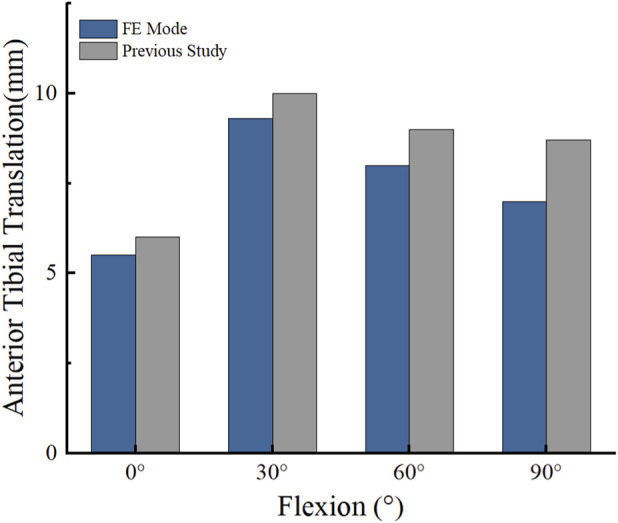
Comparison between previous studies and the present study: tibial anterior translation distance at different knee flexion angles.

The effectiveness of the finite element models was evaluated using two distinct loading protocols. Under a vertical load of 1000 N, the contact stress on the femoral cartilage surface measured 2.401 MPa, while the tibial cartilage registered 2.69 MPa. These findings demonstrated robust quantitative agreement with previous experimental data, yielding a maximum relative error of approximately 7.6% (Li et al., 2001) (Figure 5). Furthermore, in the anterior drawer experiment with a forward thrust of 134 N, the tibia advanced by 5.50–9.3 mm at various knee flexion angles. The maximum absolute discrepancy in anterior translation was less than 1.5 mm across all flexion angles. Despite these minor differences in absolute values—which primarily stem from individual anatomical variations of our specific subject model compared to average cadaveric specimens, as well as the inherent linear elastic material simplifications—the observed trend closely mirrored previous experimental and computational studies (Kang et al., 2018b) (Figure 6).

### Effects of coronal component alignment on tibial plateau stress

**FIGURE 7:**
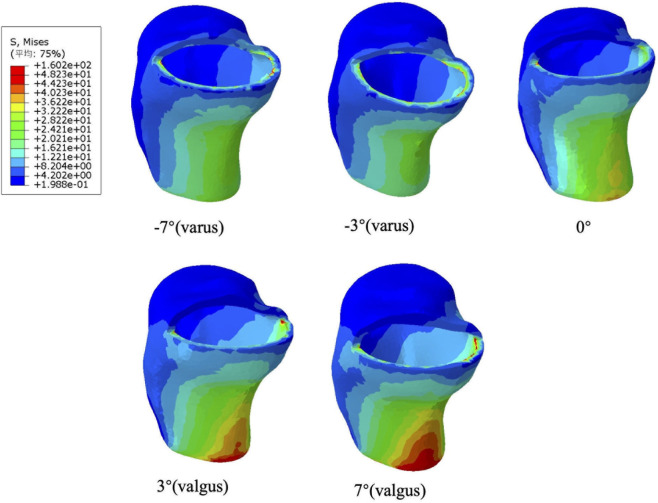
Distribution of maximum Von Mises stress on the tibial plateau under different coronal alignment angles (−7° varus, −3° varus, 0°, 3° valgus and 7° valgus).

The biomechanical reactions of unicompartmental models under gait loading at different coronal angles (−7°, −3°, 0°, 3°, and 7°) are shown in Figure 7. In the neutral position of the unicompartmental knee arthroplasty model, the maximum stress on the posterior medial side of the tibial plateau is 19.01 MPa post-gait loading. As the varus angle increases in the unicompartmental models, the maximum stress on the posterior cortex of the tibial plateau initially decreases and then increases. At an inversion angle of 3°, the minimum stress value is 18.06 MPa. Conversely, when the inversion angle reaches 7°, the maximum stress value rises to 20.23 MPa. Additionally, as the eversion angle increases, the stress on the posterior cortical bone significantly rises, peaking at 48.15 MPa when the eversion angle is 7°.

**FIGURE 8:**
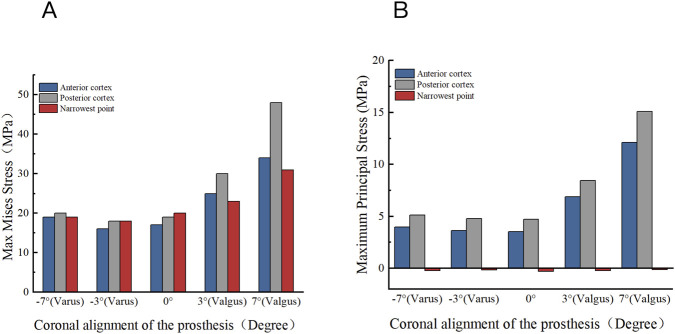
Stress distribution on the tibial plateau under different coronal alignment angles. **(A)** Maximum Von Mises stress. **(B)** Maximum Principal stress.

Figure 8A illustrates the maximum von Mises stress distribution on the tibial plateau in relation to knee joint prostheses under various coronal alignment abnormalities. As the eversion angle of the unicompartmental prosthesis changes, similar stress variations occur at the anterior, posterior, and contraction points on the tibial side. Notably, the maximum stress is primarily concentrated in the posterior medial tibial cortex. At an inversion angle of 3°, the stress on the tibial plateau is minimized. Furthermore, principal stress analysis (Figure 8B) revealed critical insights into the fracture initiation mechanism. Under neutral alignment, the Maximum Principal Stress (tensile) at the posterior cortex was 4.73 MPa. However, this tensile stress surged exponentially with excessive valgus alignment, peaking at 15.1 MPa at 7° valgus. In contrast, the narrowest point of the tibia predominantly experienced compressive stress (ranging from −0.14 MPa to −0.30 MPa). These findings confirm that extreme valgus loading concentrates highly destructive tensile forces on the posterior cortex.

### Effects of resection depth on tibial plateau stress

**FIGURE 9:**
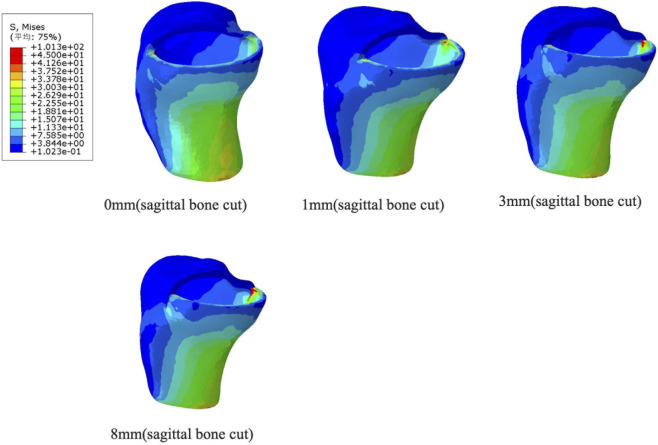
Distribution of maximum Von Mises stress on the tibial plateau under different sagittal bone cut (0 mm, 1 mm, 3 mm and 8 mm).

The medial posterior cortex of the tibia experiences the most stress due to variations in the longitudinal depth of the tibia, as seen in Figure 9. In the normal unicompartmental knee joint model, the maximum equivalent stress on this cortex is 19.01 MPa. An over-cut of 1 mm increases the maximum equivalent stress to 28.21 MPa, and an over-cut of 3 mm raises it to 29.23 MPa. When the longitudinal cut reaches 8 mm, the equivalent stress on the posterior cortex peaks at 45.10 MPa.

**FIGURE 10:**
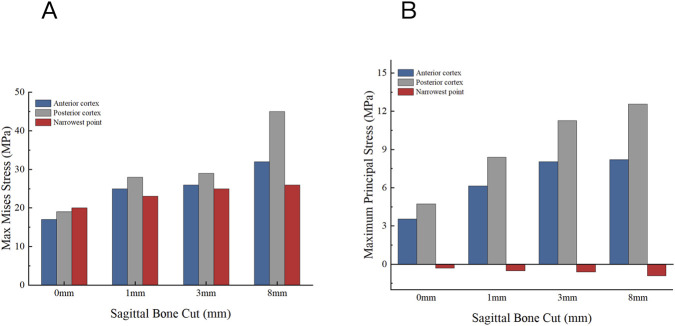
Stress distribution on the tibial plateau under different sagittal bone cuts. **(A)** Maximum Von Mises stress. **(B)** Maximum Principal stress.

The distribution of maximal von Mises stress in the tibial plateau for different longitudinal tibial incision depths is shown in Figure 10A. The posterior medial cortex of the tibial plateau shows the biggest change in tibial stress as the incision depth increases. Consistent with the structural loading patterns, the tensile fracture risk increased significantly with deeper sagittal bone cuts. As shown in Figure 10B, the Maximum Principal Stress at the posterior cortex elevated rapidly from 8.39 MPa (1 mm cut) to 12.56 MPa (8 mm cut), drastically compromising the cortical integrity and predisposing the tibia to split fractures.

### Effects of tibiofemoral component positioning on tibial plateau stress

**FIGURE 11:**
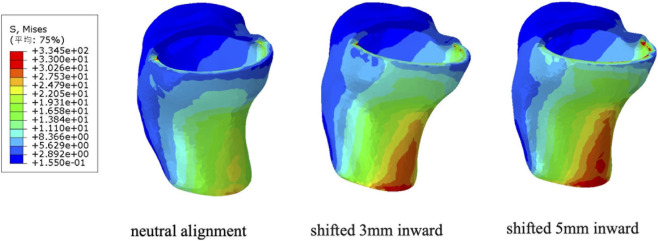
Distribution of maximum Von Mises stress on the tibial plateau under different femoro-tibial prosthetic alignment (neutral alignment, 3 mm medial displacement of femoral component relative to tibial baseplate and 5 mm medial displacement).

The effects of shifting the femoral prosthesis’s position in relation to the tibial prosthesis during gait loading on tibial stress are shown in Figure 11. With the femoral prosthesis centrally positioned, the maximum stress on the posterior tibial cortex is 19.01 MPa. Shifting the femoral prosthesis by 3 mm increases the maximum stress on the posterior cortex to 25.13 MPa. A 5 mm shift results in a maximum stress of 33.24 MPa on the posterior cortex.

**FIGURE 12:**
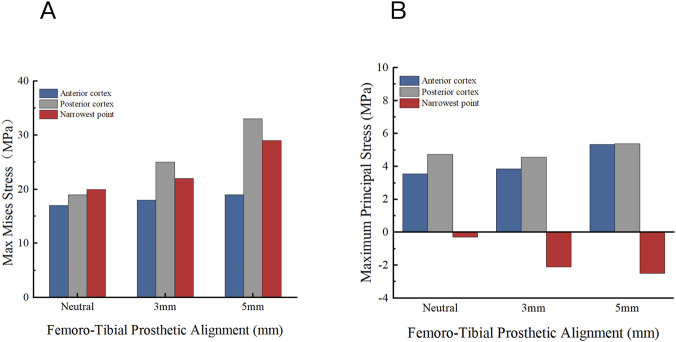
Stress distribution on the tibial plateau under different femoro-tibial prosthetic alignments. **(A)** Maximum Von Mises stress. **(B)** Maximum Principal stress.

Figure 12A illustrates the distribution of maximum Von Mises stress on the tibial side as the femoral prosthesis moves relative to the tibial prosthesis. As the femoral prosthesis shifts, the maximum stress in the anterior cortex remains relatively unchanged, whereas the posterior cortex experiences the most significant variation. Medial shifting of the tibial component also elevated the localized tensile stress at the posterior cortex, reaching 5.38 MPa at a 5 mm shift (Figure 12B). Concurrently, the compressive stress at the narrowest point intensified to −2.50 MPa, indicating a complex localized stress gradient caused by prosthetic overhang.

## Discussion

This study investigated the biomechanical risk factors for tibial plateau fractures following Oxford unicompartmental arthroplasty by developing a finite element model under gait loading. The findings revealed that variations in the coronal alignment and relative positioning of the femoral and tibial components post-surgery increase stress on the tibial side during gait loading, thereby elevating the risk of tibial plateau fractures. Additionally, the depth of the longitudinal incision on the tibial side during the procedure significantly heightens stress on the tibial cortex. Based on these findings, we recommend that during surgery, the Oxford unicompartmental prosthesis be positioned at 3° varus in the coronal plane, the femoral prosthesis be positioned no more than 3 mm from the tibial prosthesis, and the longitudinal incision on the tibial side be limited to a depth of 3 mm. These adjustments can substantially reduce stress on the tibial side and decrease the likelihood of tibial plateau fractures following Oxford unicompartmental knee replacement.

Our research indicates that, irrespective of prosthesis position changes, the maximum stress on the tibial cortical bone after unicompartmental arthroplasty is primarily concentrated in the posterior medial cortex of the tibial plateau, followed by the tibial constriction point, with the least stress in the anterior medial cortex. When the prosthesis is positioned coronally and everted by 7°, the maximum stress in the posterior cortex is 2.5 times greater than when it is everted by 3°. Additionally, if the femoral prosthesis is shifted 5 mm relative to the tibial prosthesis, the maximum stress in the posterior cortex increases to 1.7 times that of the neutral position. At an 8 mm longitudinal section of the tibia, the maximum stress rises to 2.3 times the neutral position. Outward placement can lead to stress concentration, particularly in the posterior cortex, and reduce the effective supporting bone volume. Notably, the stress increase due to eversion is greater than that caused by inversion, indicating a higher fracture risk from poor eversion alignment. Moreover, making an excessively deep longitudinal incision during surgery can substantially weaken the posterior tibial cortical bone, which bears the primary stress and can become the fracture’s initiation point. This finding aligns with the post-cortical stress concentration phenomenon observed in finite element analysis. Additionally, the anatomical structure of the proximal tibia means that for the medial Oxford unicompartmental prosthesis, any misalignment between the femur and tibial prosthesis in the medial and lateral directions results in eccentric loading, significantly elevating the maximum stress on the posterior cortical bone. By implementing a dual-metric assessment incorporating both von Mises and Maximum Principal stresses, our study successfully differentiated between general load-bearing capacity and specific tensile failure risks. The principal stress data clearly highlighted that split fractures following UKA are mechanically initiated by excessive tensile forces concentrated at the posterior cortex—forces that are exponentially amplified by severe valgus alignment and excessive sagittal bone cutting. Our findings align with prior research (Innocenti et al., 2016; Kang et al., 2018a; Inoue et al., 2016). Inoue et al. (Inoue et al., 2016) developed a finite element model of the tibia and discovered that post-UKA, stress on the tibial cortex increased as the prosthesis shifted from inward to outward. Interestingly, they found that a 6° varus alignment of the tibial prosthesis resulted in the least stress on the cortex, a conclusion that differs slightly from our finding of an optimal 3° varus alignment. This discrepancy is largely attributable to methodological distinctions in prosthesis design and loading protocols. Inoue et al. evaluated fixed-bearing prostheses under static loading conditions. In contrast, our study simulated a mobile-bearing design (Oxford Phase III) subjected to complex dynamic gait cycles. The mobile-bearing insert allows for micro-kinematic adaptations during dynamic ambulation, thereby altering the optimal stress-transfer pathway. Innocenti et al. (Innocenti et al., 2016) also confirmed that the optimal coronal position for the UKA tibial plateau prosthesis is either neutral or with a 3° inversion. However, these earlier studies primarily used static loads and focused on fixed platform unicompartmental prostheses. Our study simulated changes in the tibial cortex following Oxford unicompartmental arthroplasty under dynamic gait loading. During the peak loading phase of standing (10%-20% of the gait cycle), we observed that poor inversion and varus alignment significantly increased stress concentration, which may explain the mechanism behind fractures occurring after early weight-bearing in clinical practice. An important consideration in tibial treatment is the risk of damaging the posterior cortex due to excessively deep longitudinal sawing. Rudol et al. (Rudol et al., 2007) documented a case of a tibial plateau fracture resulting from such sawing. Furthermore, cadaver experiments by Clarius et al. (Clarius et al., 2010) demonstrated that the fracture load of a tibial plateau extended by 10° through longitudinal sawing decreased by approximately 30%. Currently, there is limited research on the positional deviation between the Oxford unicompartmental femoral prosthesis and the tibial prosthesis. Although the design of the movable platform can somewhat accommodate slight positional deviations, this study reveals that when the femoral prosthesis shifts inward by more than 3 mm, it causes excessive stress concentration on the tibial cortex. Unlike most previous studies, which applied static loads, this study simulated dynamic load changes throughout the entire gait cycle, allowing us to observe the evolution of stress over time. These dynamic analysis results are crucial for understanding the timing of clinical fractures. Most early tibial fractures reported in the literature after UKA occur approximately 3 months after patients start partial weight-bearing walking (Burger et al., 2022; Mohammad et al., 2024), aligning with the timing of stress peaks we observed under gait loading. Patients with poor prosthesis positioning are particularly susceptible, as cumulative micro-injuries from repetitive gait loads in stress concentration areas may eventually result in fatigue fractures. Clinically, it is crucial to advise patients to avoid activities that may generate high loads, such as rapid walking and sudden turns, during the early postoperative period, especially for those with suboptimal prosthesis positioning (Eickhoff et al., 2023).

Currently, biomechanical research on tibial plateau fractures following UKA surgery is limited. Inoue et al. (Inoue et al., 2016) analyzed the impact of the prosthesis’s coronal alignment on the tibial side using a finite element model, but did not develop a comprehensive model for unicompartmental displacement. Clarius et al. (Clarius et al., 2010) conducted cadaver experiments to evaluate tibial strain at various longitudinal cutting depths, but precise parameter control was challenging. Previous studies primarily focused on stress changes in polyethylene gaskets, applying a single load without thoroughly investigating stress distribution on the tibial side, particularly at the narrowing point. This study first applied gait loads to more accurately simulate clinical conditions. It not only examined stress peaks on the tibial side but also analyzed stress distribution patterns and concentration points, offering detailed insights into the fracture mechanism. Importantly, the study designed a prosthesis for the Oxford unicompartmental knee, an active platform type, providing crucial biomechanical evidence to reduce postoperative tibial plateau fracture complications.

This study has certain limitations. First, the finite element model relies on a series of simplified assumptions, such as the isotropic nature of material properties and the fully fixed bone-prosthesis interface. Specifically, biological tissues and the UHMWPE insert were simplified as linear elastic materials. While this omits their inherent non-linear behaviors, it is a justified engineering simplification. According to Saint-Venant’s principle, highly localized contact deformations do not significantly alter the macroscopic stress patterns in the distant cortical shell, which was the primary focus of our fracture risk assessment. This approach also facilitated convergence during the explicit dynamic analysis of the complete gait cycle. Furthermore, the model validation was performed exclusively on the intact knee joint rather than the post-implant UKA configuration. While an explicit post-UKA validation is ideal, it is hindered by the scarcity of *in vivo* experimental data directly matching this specific prosthesis under complex gait loading. Validating the intact knee is a standard prerequisite in computational biomechanics to ensure the baseline physiological accuracy of ligaments and bone structures before surgical alterations. Consequently, the absolute stress values should be interpreted with caution, whereas the relative stress variations among different surgical parametric groups remain highly reliable. These simplifications may affect the model’s absolute accuracy. However, since the study primarily focuses on relative comparisons (stress differences under various parameter conditions), the impact of these simplifications on the main conclusions should be minimal. Second, the study did not account for the restrictive effects of soft tissue structures, like joint capsules and muscles. Thirdly, while the use of a healthy 38-year-old model may limit the direct extrapolation of absolute stress values to the elderly osteoporotic population, the biomechanical trends identified in this parametric study remain consistent. Future research incorporating varied bone densities would further refine these clinical recommendations. Lastly, only gait loads were considered in this study, while daily activities such as squatting and stair navigation were not examined.

## Conclusion

This study employed systematic finite element analysis of the Oxford unicompartmental system to identify three key surgical parameters that significantly impact the risk of tibial plateau fractures post-UKA: the coronal alignment angle, the sagittal bone cut, and the relative position of the femoral and tibial prostheses. The results demonstrate that prosthesis malalignment, particularly excessive valgus, and excessively deep longitudinal tibial incision are primary contributors to elevated stress on the tibial plateau. Furthermore, femoral-tibial prosthesis mismatch further exacerbates the stress concentration effect. Importantly, these parameters exhibit nonlinear interactions, and certain high-risk parameter combinations can significantly elevate fracture risk. These findings emphasize the critical importance of precise surgical techniques and provide valuable biomechanical evidence for preoperative planning, intraoperative procedures, and postoperative management. By optimizing these key surgical parameters and implementing appropriate risk stratification strategies, the incidence of tibial plateau fractures following UKA can be effectively reduced.

## Data Availability

The original contributions presented in the study are included in the article/supplementary material, further inquiries can be directed to the corresponding author.
